# The TRACK-PD study: protocol of a longitudinal ultra-high field imaging study in Parkinson’s disease

**DOI:** 10.1186/s12883-020-01874-2

**Published:** 2020-08-05

**Authors:** A. F. Wolters, M. Heijmans, S. Michielse, A. F. G. Leentjens, A. A. Postma, J. F. A. Jansen, D. Ivanov, A. A. Duits, Y. Temel, M. L. Kuijf

**Affiliations:** 1 Department of Neurology, Maastricht University Medical Centre, P.O. Box 5800, 6202 AZ Maastricht, The Netherlands; 2 School for Mental Health and Neuroscience, EURON, Maastricht University, P.O. Box 616, 6200 MD Maastricht, The Netherlands; 3 Department of Psychiatry, Maastricht University Medical Centre, P.O. Box 5800, 6202 AZ Maastricht, The Netherlands; 4 Department of Radiology and Nuclear Medicine, Maastricht University Medical Centre, P.O. Box 5800, 6202 AZ Maastricht, The Netherlands; 5 Department of Cognitive Neuroscience, Faculty of Psychology and Neuroscience, Maastricht University, P.O. Box 616, 6200 MD Maastricht, The Netherlands; 6 Department of Medical Psychology, Maastricht University Medical Centre, P.O. Box 5800, 6202 AZ Maastricht, The Netherlands; 7 Department of Neurosurgery, Maastricht University Medical Centre, P.O. Box 5800, 6202 AZ Maastricht, The Netherlands

**Keywords:** Cohort studies, Parkinson’s disease, Ultra-high field MRI, Biomarkers

## Abstract

**Background:**

The diagnosis of Parkinson’s Disease (PD) remains a challenge and is currently based on the assessment of clinical symptoms. PD is also a heterogeneous disease with great variability in symptoms, disease course, and response to therapy. There is a general need for a better understanding of this heterogeneity and the interlinked long-term changes in brain function and structure in PD. Over the past years there is increasing interest in the value of new paradigms in Magnetic Resonance Imaging (MRI) and the potential of ultra-high field strength imaging in the diagnostic work-up of PD. With this multimodal 7 T MRI study, our objectives are: 1) To identify distinctive MRI characteristics in PD patients and to create a diagnostic tool based on these differences. 2) To correlate MRI characteristics to clinical phenotype, genetics and progression of symptoms. 3) To detect future imaging biomarkers for disease progression that could be valuable for the evaluation of new therapies.

**Methods:**

The TRACK-PD study is a longitudinal observational study in a cohort of 130 recently diagnosed (≤ 3 years after diagnosis) PD patients and 60 age-matched healthy controls (HC). A 7 T MRI of the brain will be performed at baseline and repeated after 2 and 4 years. Complete assessment of motor, cognitive, neuropsychiatric and autonomic symptoms will be performed at baseline and follow-up visits with wearable sensors, validated questionnaires and rating scales. At baseline a blood DNA sample will also be collected.

**Discussion:**

This is the first longitudinal, observational, 7 T MRI study in PD patients. With this study, an important contribution can be made to the improvement of the current diagnostic process in PD. Moreover, this study will be able to provide valuable information related to the different clinical phenotypes of PD and their correlating MRI characteristics. The long-term aim of this study is to better understand PD and develop new biomarkers for disease progression which may help new therapy development. Eventually, this may lead to predictive models for individual PD patients and towards personalized medicine in the future.

**Trial registration:**

Dutch Trial Register, NL7558. Registered March 11, 2019.

## Background

Parkinson’s disease (PD) is the second most common neurodegenerative disorder after Alzheimer’s disease and is characterized by motor symptoms such as bradykinesia, rigidity and tremor [1–3]. Patients with PD also experience a broad spectrum of non-motor symptoms such as anxiety, depression, pain and autonomic dysfunction, making the disease a typical example of a neuropsychiatric disorder. The diagnosis of PD is currently based on the assessment of clinical symptoms and their course over time. However, the early diagnosis of PD can be challenging since its presentation is heterogeneous and mild symptoms are often not immediately recognised. Clinico-pathological research shows that the error rate for a clinical diagnosis of PD can be as high as 24%, even in specialized centres [4].

Recently, clinical criteria have been revised for the diagnosis of PD [3] and several subtypes of PD are being recognised [5, 6]. Currently a Movement Disorder Society (MDS) task force is mandated to review the evidence for these subtypes and propose a subtype classification system. The different subtypes of PD might be influenced by a combination of environmental and genetic factors [7]. However, the underlying aetiology of the clinical heterogeneity in PD is not well understood [8]. This is why the National Institutes of Health (NIH) pointed out that obtaining more insight in this heterogenous nature and defining the different PD subtypes, is one of the top three research priorities in PD [9].

In clinical practice, Magnetic Resonance Imaging (MRI) is currently used as a method to exclude other potential causes of parkinsonian symptoms. To date, it is impossible to diagnose a patient with PD based on MRI characteristics. However, over the past years it has become well-established that MRI may serve as a valuable method in the diagnostic work-up of PD [10]. Early indicators, such as signal loss of the nigrosome-1 area on iron-sensitive MR Images and reduced volume and signal intensity of the substantia nigra on neuromelanin-sensitive images, have been described in PD [11–14]. Additionally, functional MRI (fMRI) techniques can display changes related to specific symptoms in PD [15, 16]. With the emergence of ultra-high-field scanners (7 T and above) submillimetre anatomical information can be obtained. Compared with 3 T MRI, ultra-high-field MRI at 7 T provides an increased spatial resolution and a higher signal-to-noise ratio enabling a potential higher degree of diagnostic detail [10].

The primary objective of this study is to identify early and subtle MRI changes in PD patients which distinguish them from the healthy population and to create a reliable tool for the early diagnosis of PD based on these differences. Secondary objectives are to detect whether different clinical phenotypes of PD patients also show different imaging characteristics and to design a prognostic tool for individual PD patients by correlating specific MRI characteristics to clinical phenotype, genetic characteristics and progression of symptoms. Moreover, this large longitudinal ultra-high field imaging study may eventually also find new imaging biomarkers for disease progression that could be valuable for the development and evaluation of new therapies in PD. In addition, the database will serve as a biobank for further related research.

## Methods

This is a longitudinal observational study in PD patients and healthy controls (HC). The TRACK-PD study (www.trackpd.nl) will assess the structural and functional characteristics of PD patients on ultra-high field 7 T MRI. The study has been registered at the Dutch Trial Register (www.trialregister.nl) with identification number NL7558.

### Participants

In this study, 130 participants with PD will be recruited from the PD population visiting the movement disorder clinic of the Department of Neurology of the Maastricht University Medical Centre and other collaborating hospitals. In addition, we will use other media, such as websites, social media and patient meetings to recruit patients. 60 HC participants will be recruited through advertisements in the hospital and university.

### Inclusion and exclusion criteria

Participants are eligible for participation in this study if they meet the following criteria: 1) All patients have to be diagnosed with PD by a neurologist, within the last 3 years before inclusion. 2) A score of ≥24 on the Montreal Cognitive Assessment (MoCA) at baseline. 3) Able to read and understand Dutch. 4) 18 years of age or older. 5) Providing written informed consent.

Participants with advanced cognitive impairment, defined as a score of < 24 on the Montreal Cognitive Assessment (MoCA), or a diagnosis of dementia according to the fifth edition of the Diagnostic and Statistical Manual of Mental Disorders (DSM 5, [17]) criteria at baseline, will be excluded from participation. The presence of a clear diagnosis of neurodegenerative diseases other than PD is also an exclusion criterion. Lastly, potential participants cannot take part if there are any contra-indications for a 7 T MRI scan, such as claustrophobia, permanent makeup or the presence of incompatible metallic devices in their body. These exclusion criteria are also in place for the HC group.

### Study procedure

This is a longitudinal observational study in which all participants will be tracked for 4 years. All data will be collected in one academic hospital in the Netherlands (Maastricht University Medical Centre). A 7 T brain MRI will be conducted at baseline, after 2 years and after 4 years. Basic clinical and demographic information, such as age, sex, handedness, disease duration, and the total levodopa equivalent daily dose (LEDD, [18]) will also be collected. Motor, cognitive, autonomic and neuropsychiatric symptoms will all be assessed by validated questionnaires and rating scales as summarized in Table 1. The assessments have been aligned with other national and international PD cohort studies, to permit future cross validation studies [19, 20]. Wearable sensors measuring movement will be applied at each wrist and at the chest during the assessment days. In addition to the tests described above, a blood sample from all participants will be collected at baseline, which will be used for genetic and epigenetic testing on genes related to PD. Providing a blood sample is not mandatory for participation.

**Table 1:** Overview of included study measures and scales in the TRACK-PD study

Method	Outcome	Scales	Visit 1 (Baseline)	Visit 2 (2 Years)*˟*	Visit 3 (4 Years)*˟*
Assessed by trial assessor	Motor functioning in ‘ON’ state	MDS-UPDRS III (including H&Y stage)	X*	X*	X*
		MDS-UPDRS IV	X*	X*	X*
	Neuropsychological symptoms	MoCA	X	X	X
		Phonemic and semantic fluency	X	X	X
		15 Words Test	X	X	X
		Benton Judgment of Line Orientation	X	X	X
		Letter Number Sequencing	X	X	X
		Symbol Digit Modalities Test	X	X	X
		MDS-UPDRS I	X*	X*	X*
	Demographics and lifestyle	Medical history	X	X	X
		Medication	X	X	X
	Biospecimen	EDTA Plasma (DNA)	X		
		Pax Gene (RNA)	X		
	Brain structure and function	Resting-state functional MRI	X	X	X
		Structural MRI (T1, T2*, neuromelanin, DWI)	X	X	X
Wearable sensors	Motor parameters	IMU including 3-axis accelerometer and 3-axis gyroscope	X	X	X
Self-reported patient questionnaires	Neuropsychiatric symptoms	BDI	X	X	X
		QUIP-RS	X	X	X
		PAS	X	X	X
	Quality of life	PDQ-8	X*	X*	X*
	Autonomic symptoms	SCOPA-AUT	X	X	X
	Sleep disorders	RBDQ	X	X	X
	Various	MDS-UPDRS II	X*	X*	X*

*BDI* Beck Depression Inventory, *HC* healthy controls, *H&Y* Hoehn and Yahr scale, *IMU* Inertial Measurement Unit, *MDS-UPDRS* Movement Disorders Society Unified Parkinson Disease Rating Scale, *MoCA* Montreal Cognitive Assessment, *NPA* Neuropsychological assessment, *PAS* Parkinson Anxiety Scale, *PD* Parkinson’s disease patients, *PDQ-8* Parkinson’s Disease Questionnaire, *QUIP-RS* Questionnaire for Impulsive-Compulsive Disorders in Parkinson’s disease, *RBDSQ* REM-sleep behaviour disorder screening questionnaire, *SCOPA-AUT* Scales for Outcomes in Parkinson’s Disease - Autonomic dysfunction questionnaire

˟ ± 60 days; * Only for PD patients

### Clinical assessments

#### Motor assessment

Motor functions will be assessed with the unified Parkinson’s Disease rating scale (MDS-UPDRS) and the Hoehn and Yahr scale (H&Y). The H&Y is the most commonly used scale to estimate the global disease stage of PD patients [21]. The MDS-UPDRS consists of four parts, assessing both non-motor and motor disabilities in PD [22]. All four parts will be assessed by trained and certified investigators. At baseline, the UPDRS part III will also be used to check whether patients meet the MDS clinical diagnostic criteria for parkinsonism [3]. The motor evaluation is performed in medication ‘ON’ state.

Furthermore, wearable sensor data will be collected on all testing days. The wearables contain both an accelerometer and a gyroscope and are described in more detail elsewhere [23]. During each testing day, the wearables will be put on after signing the informed consent form and will be removed while preparing the participant for the 7 T MRI. The wearables will be applied to the wrists by using comfortable straps. A hanger will be used for the chest wearable.

#### Autonomic assessment

The Scales for Outcomes in Parkinson’s Disease - Autonomic dysfunction (SCOPA-AUT) questionnaire will be applied to assess autonomic functions in all participants. This questionnaire consists of 25 items assessing several different autonomic symptoms in patients with PD. It is a reliable and valid questionnaire for the evaluation of autonomic dysfunction in PD [24].

#### Neuropsychological assessment

At baseline, global cognitive function is assessed with the MoCA, which is a 30 point screening tool, including several cognitive domains [25]. If, at baseline the MoCA score is ≥24, the participant can continue the study protocol and a full neuropsychological assessment will be performed. If the MoCA score drops below 24 during follow-up, participants will remain in the study. Neuropsychological assessment consists of the following standard tests: 1. The ‘Phonemic and Semantic Fluency Test’ for executive function [26]; 2. The ‘Auditory Verbal Learning Test’ for memory [27]; 3. The ‘Benton Judgment of Line Orientation’ for visuospatial function [28]; 4. The ‘Symbol Digit Modalities Test’ for mental speed and attention [29]; and 5. The ‘Letter Number Sequencing Test’ for working memory [30].

#### Neuropsychiatric assessment

In addition, several questionnaires will assess psychiatric symptoms. The ‘Beck Depression Inventory’ (BDI), a 21-item questionnaire, is used to assess depression [31]. The Parkinson Anxiety Scale (PAS) [32] is used to assess anxiety. The ‘Questionnaire for Impulsive-Compulsive Disorders’ in Parkinson’s disease (QUIP-RS) will assess impulsive and compulsive behaviours [33]. The Parkinson’s Disease Questionnaire (PDQ-8) assesses quality of life of PD patients [34]. For each questionnaire the total sum score will be calculated.

#### REM-sleep behaviour disorders

Symptoms related to REM-sleep behaviour disorders will be assessed by using the ‘REM-sleep behaviour disorder screening questionnaire’ (RBDSQ). This questionnaire is a 13-item screening tool for the detection of REM sleep behaviour disorders [35]. A total score will be calculated.

#### Genetic testing

Several contributing factors for PD risk, onset, and progression of symptoms have been associated with genetic variants. Especially in early-onset PD, strong penetrant mutations have been found. Possible new MRI correlates with known genetic variants could be valuable in further understanding the genetic basis and progression of PD. However, more than 20 mutations in genes have been associated with PD onset and more than 90 independent risk-associated variants in genome-wide association studies have been reported [36]. Blood samples of the participants will be tested on common genetic variants and stored in a database for further epigenetic testing, including the examination of DNA methylation patterns [37]. Drawing of the blood samples will be performed only by researchers or research nurses who are certified to do this. From every participant two ethylenediamine tetra-acetic acid buffered (EDTA) tubes with a size of 4.5 ml each and two PAXgene® tubes with a size of 2.5 ml each will be collected. These blood samples will be transferred to the BioBank of Maastricht University Medical Centre, were they will be stored at − 80 °C.

### MRI acquisition

Participants will be scanned on a 7 T MRI scanner (Magnetom, Siemens, Erlangen, Germany) equipped with a Nova Medical 32-channel head coil. Dielectric pads will be applied to enhance the signal in the temporal brain regions [38]. In PD patients, the MRI will be carried out while they are in the ON-medication state in order to reduce the possibility of motion artefacts due to tremor and to reflect clinical practice. During the scan session participants will also watch a movie to reduce movements. Cardiac and respiratory physiological signals will be measured synchronized with the scan start.

**Fig. 1:**
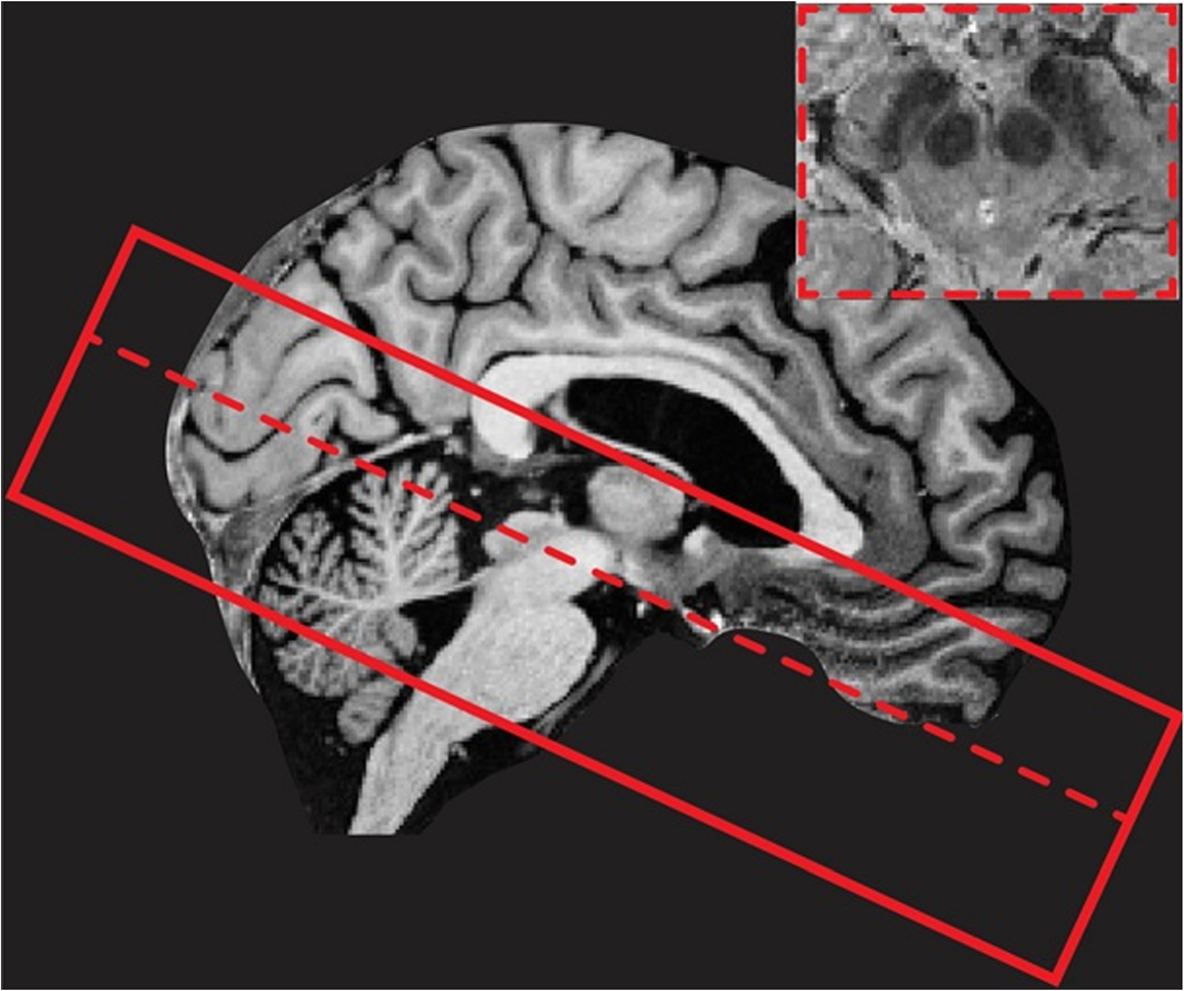
Visualisation of the multi-echo GRE field-of-view (FoV) with 4.8 cm coverage. The FoV is placed perpendicular to the brainstem. The inferior border of the FoV is positioned at the bottom of the 4th ventricle. Furthermore, an example of the multi-echo GRE transverse brain stem slice at the substantia nigra is displayed in the right corner

**Fig. 2:**
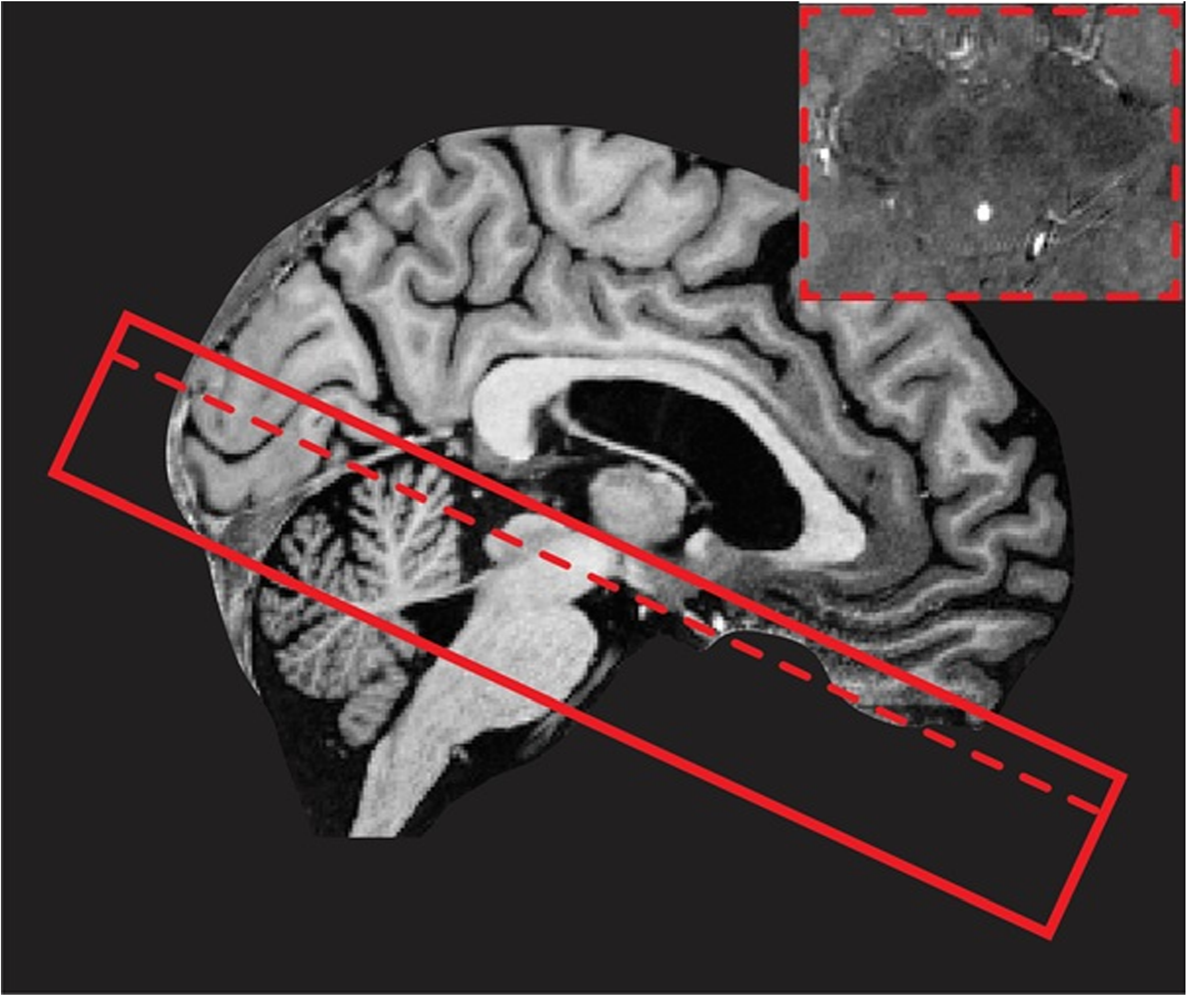
Visualisation of the magnetization transfer-weighted TFL (MTW TFL) field-of-view (FoV) with 3 cm coverage. The FoV is placed perpendicular to the brainstem. It covers the area between the upper part of the mesencephalon and the lateral recess of the 4th ventricle. Furthermore, an example of the MTW TFL transverse brain stem slice at the substantia nigra is displayed in the right corner

A localizer sequence will be acquired for optimal planning. B0 and B1 mapping and shimming will be performed to correct for field inhomogeneities. The scan protocol consists of 1) a whole-brain MP2RAGE (Magnetization Prepared 2 Rapid Acquisition Gradient Echoes) scan with an acquisition time of 10:57 min, resulting in a T1-weighted image and a quantitative T1 map. This MP2RAGE is combined with a SA2RAGE (Saturation Prepared with 2 Rapid Gradient Echoes) scan of 2:40 min, to eliminate any B1-related biases from the results, which can reduce the accuracy of the measurements [39], 2) A multi-echo GRE, 4.8 cm coverage, acquisition time 7:42 min, used to provide susceptibility (T2*)-weighted images and T2* maps. In addition, quantitative susceptibility maps can be reconstructed from the same acquisition. This scan is sensitive for measuring variations in iron concentration (Field of View (FoV) is shown in Fig. 1), 3) A magnetization transfer-weighted TFL (MTW TFL), 3 cm coverage, acquisition time 4:38 min, which can be used to visualise myelination or nuclei containing neuromelanin such as the substantia nigra and locus coeruleus [40] (FoV is shown in Fig. 2), 4) A whole-brain diffusion weighted scan along 66 random directions with an average b-value of 2000s/mm^2^ mixed with six B0-volumes and one additional B0-volume and five diffusion weighted volume recorded with opposite phase-encoding direction, acquisition time 9:48 min, 5) A whole brain resting-state fMRI scan with 280 volumes and an additional five volumes recorded with reverse phase-encoding direction. Acquisition time for the resting-state fMRI is 11:12 min. During the resting-state fMRI scan participants are instructed to focus on a crosshair projected on a screen while letting their mind wander and not to think about anything in particular. In total the scan protocol takes a little less than 60 min and details can be found in Table 2.

**Table 2:** Technical details of the MRI protocol used for the TRACK-PD study

Weighting	Sequence	TE (ms)	TR (ms)	TI (ms)	Flip angle (°)	FoV (mm)	Resolution (mm^3^) (x-y-z)	Slices	Orientation
T1	MP2RAGE	2.51	5000	900, 2750	5 and 3	208	0.65 × 0.65 × 0.65	240	Sagittal
	SA2RAGE	0.78	2400	58, 1800	4 and 10	256	2.0 × 2.0 × 2.0	88	Sagittal
T2*	GRE	2.49, 6.75, 13.50, 20.75	33	–	12	204	0.5 × 0.5 × 0.5	96	Axial
Neuromelanin	MTW TFL	4.08	538	–	8	192	0.4 × 0.4 × 0.5	60	Axial
Diffusion	EPI	60.6	7000	–	90	192	1.5 × 1.5 × 1.5	80	Axial
fMRI	BOLD	18.6	2000	–	70	200	1.25 × 1.25 × 1.25	92	Axial

*MP2RAGE* Magnetization Prepared 2 Rapid Acquisition Gradient Echoes. *SA2RAGE* Saturation Prepared with 2 Rapid Gradient Echoes), *GRE* Gradient Echo, *MTW TFL* Magnetization transfer weighted TFL, *EPI* Echo Planar Imaging, *BOLD* Blood oxygen level dependent, *TE* Echo time, *TR* Repetition time, *TI* Inversion Time, *FoV* Field of view

### Sample size calculation

Previous longitudinal MRI studies in PD have included a variety of sample sizes, but most studies contain about 15 to 25 patients and HC [41–43]. One larger longitudinal 3 T MRI study, the ICICLE-PD, recruited 105 PD patients and 37 matched HC and followed their participants for 18 months [44]. So far, no longitudinal 7 T studies nor 7 T MRI studies combining multiple MRI sequences in PD patients have been published. However, several cross-sectional 7 T MRI studies have been performed, all consisting of relatively small patient groups varying from 13 to 36 PD patients [45–49].

A sample size calculation was based on the effect size of a study that evaluated the dorsal nigral hyperintensity sign in PD and HC at T2* weighted 7 T images [47]. Our power analysis with a significance level of α = 0.05 and power of 0.80 was performed with G*Power (version 3.1.9.4) [50]. Based on this analysis a total sample size of 102 participants, 51 PD and 51 HC, is needed to detect a significant difference between these two groups. Due to an expected loss to follow-up of about 10 % for HC, we decided to include 60 HC patients instead of 51. For our secondary objectives we will also perform subgroup analysis for PD patients. To assure a sufficient amount of PD patients for this subgroup analysis, we decided to double the amount of PD patients to 102. Furthermore, the drop-out rate in the PD group is expected to be higher compared to the HCs, due to an increase in disease burden over time. With an expected loss to follow-up of 20 %, the PD group should include 130 participants.

### Patients and public involvement

This study has been designed in collaboration with the Dutch Parkinson society. This patient organisation has a scientific advisory board that comments on and contributes to research proposals from a patient’s perspective. They will remain involved in the study during the inclusion and follow-up period. Throughout the study, we aim to stay in close contact with all participants. They will be kept informed about the study progress with newsletters and updates on the study website. Moreover, when problems or questions occur, participants can easily contact the study team by e-mail or phone. In this way we intend to minimise the loss to follow-up.

### Data collection and management

The data collected in this study will be stored in a pseudonymised manner. All patient data will be linked to a unique participant number. Two separate databases are in place. Source data of the participants will be kept in a password protected, secure database, which is only accessible by the research team, the health inspector, the data monitor (of the CTCM (Clinical Trial Centre Maastricht)) and members of the medical ethics committee. A second database will be used to store all experimental data and participant numbers. The anonymous data will only be used for research purposes and publications or communications. The handling of personal data will comply with the EU General Data Protection Regulation (GDPR) and the Dutch Act on Implementation of the GDPR. The final study dataset can be accessed only by the research team. Datasets will be made available for other investigators on reasonable request. All study information will be stored for 15 years. Patients who do not give consent to store their information for 15 years, cannot be included in the study.

### Data analysis

In this study we will analyse both structural and functional MRI data. Raw MRI data will be converted to BIDS NIfTI format for further processing [51, 52]. Functional and anatomical MRI images will be pre-processed using MATLAB (MathWorks, version R2018a), the FRMIB Software Library (FSL) [53] and FreeSurfer (www.freesurfer.net) [54]. Further analysis will be carried out using FSL. All multi-echo GRE, neuromelanin, DWI and functional MRI images will be coregistered with anatomical T1 weighted structural images. Segmentation of the substantia nigra (SN) and locus coeruleus (LC) is manually performed. The subareas of the SN, the pars reticulata and pars compacta ventralis and dorsalis, will also be differentiated. Volumes and signal-to-noise ratio of the SN and LC will be calculated on iron and neuromelanin sensitive images. Both quantitative and qualitative evaluation of the iron sensitive sequences will be performed. For DWI images, diffusion tensor imaging (DTI) will be applied. Furthermore, the fractional anisotropy (FA), radial diffusivity, axial diffusivity and mean diffusivity will be extracted in the SN ROIs. A whole-brain analysis using independent component analysis will be carried out for the functional MRI data. Subsequently, a graph theoretical method is performed to evaluate the topological properties in the whole brain. Finally, predefined ROIs will be assessed. A specific interest exists for regions that are part of the default mode network and fronto-parietal network.

Careful visual inspection and quantification of movement will be assessed after each processing step. Both structural and functional MRI differences between HCs and PD patients will be investigated, as well as longitudinal MRI changes in the PD brain.

Clinical and demographic variables will be taken into account as covariates, including handedness, sex, age, disease progression, dopaminergic medication, genetic characteristics, and motor, neuropsychiatric, cognitive and autonomic scores. More specifically, since dopaminergic medication influences brain connectivity patterns both in a linear and non-linear way, the LEDD will be included as a covariate in all fMRI data analysis [55, 56]. The numerical variables will be described as means, median, standard deviations and ranges. Categorical variables will be described as frequencies, percentiles, and percentages.

Demographic and disease related variables of the samples at baseline will be compared with Pearson’s chi-squared test for categorical variables (non-parametric test) and with the student’s t-test and one-way ANOVA for continuous variables. These variables will be included as covariates in the analyses where appropriate.

For our primary objective we will compare structural and functional MRI characteristics between PD patients and HC. This includes iron-sensitive, neuromelanin-sensitive, diffusion weighted and functional sequences. Structural and functional differences will be calculated by using a linear regression model for continuous variables and a logistic regression model for binary variables. Covariates will be included when needed. The false discovery rate (FDR) method for the correction of multiple comparisons will be applied where appropriate.

After performing the regression analyses for both structural and functional MRI data, we will use a receiver operating characteristics (ROC) method to compute the sensitivity and specificity of the differences found between PD and HC. Furthermore, internal validation will be carried out by using a bootstrap method. In this way we aim to detect the optimal cut-off values for structural and functional differences which distinguish PD patients from HC. Finally, the optimal combination of variables for disease prediction will be assessed by using a logistic regression model. By combining the most important variables, we aim to develop a reliable diagnostic tool with high sensitivity and specificity, that enables us to distinguish PD from HC.

For our secondary objectives, a data-driven cluster analysis will be performed based on the standardized scores of the different variables assessed in our study (motor, genetic, cognitive, autonomic, RBDSQ and neuropsychiatric variables). This enables us to identify different clinical subtypes of PD without a priori assumptions. Structural and functional MRI characteristics will be compared between these different subgroups of PD patients in the same way as described above. Also, longitudinal changes in clinical motor and non-motor symptoms and MRI characteristics will be compared between subgroups by applying a multivariate linear regression model to detect the degree of change for each variable between the PD subgroups.

Furthermore, the longitudinal clinical data (motor, genetic, cognitive, autonomic, RBDSQ and neuropsychiatric variables) of both follow-up moments will be correlated with the MRI characteristics at baseline by using a linear regression model. In this way it can be investigated if the disease course of an individual patient can be predicted based on early MRI-characteristics.

### Ethics, safety and dissemination

The study will be conducted according to the principles of the Declaration of Helsinki (Fortaleza, Brazil, 2013) and in accordance with the Medical Research Involving Human Subjects Act (WMO). All unwanted and harmful outcomes spontaneously reported by the participants, that may or not be related to this study, will be recorded. In case of a serious adverse event, the Ethics committee and relevant authorities will be notified immediately.

This study was approved by the Institutional Review Committee (IRB) of the Maastricht University Medical Centre and written informed consent will be obtained from all participants prior to inclusion. Also, participants are given the choice to provide additional consent for the use of study data and biological specimens in ancillary studies. Before consenting, all participants will be extensively informed about the study. During the study patients have the right to withdraw from the study without explanation at any time.

Monitoring of the study will be performed at random moments by employees of CTCM. Those employees are trained and certified in monitoring studies and are not in any way involved in the study.

The results of this study will be shared with clinicians and researchers through scientific conferences and publications in peer-reviewed journals. During the progress of the study preliminary analysis and data validation will be performed, new relevant findings may be published during the study. Furthermore, a summary of the results will also be shared with patients on our web page in an easily comprehensible manner.

## Discussion

The current error-rate for a reliable diagnosis of PD is unacceptable and at this moment it is unclear to what extend changes on MR imaging in PD patients are associated with the clinical deterioration over time. Ultra-high field imaging has made significant progress in recent years and has a resolution that might replace the requirement for a histologically confirmed diagnosis of PD. This new longitudinal ultra-high field 7 T MRI study in a PD cohort in which relevant clinical metrics will be obtained for 4 years, will likely improve and change our diagnostic uncertainty in PD.

Several cross-sectional and cohort studies have indicated correlations between MRI characteristics and the clinical symptoms in PD. Most convincingly, a positive correlation between the iron content of the substantia nigra pars compacta and the progression of motor symptoms in PD was demonstrated in several studies [11, 12, 41, 46]. In addition, neuromelanin sensitive T1-weighted MRI sequences of the substantia nigra pars compacta may be useful for monitoring motor complications of PD [57]. Also, functional MRI studies demonstrate an altered functional connectivity in PD compared to HC, which is most consistently found in the posterior part of the inferior parietal lobule [58]. Given these developments, the use of multiple MRI modalities and post-processing techniques such as quantitative susceptibility mapping (QSM) at higher field strengths have the potential to increase diagnostic certainty in PD. However, this has not been tested in a large longitudinal cohort study yet. Furthermore, when high-field MRI research is able to detect whether clinical deterioration or certain PD subtypes are correlated with specific MRI changes, patients can be more accurately informed about their prognosis and treatment options can be adjusted to the individual patient.

In order to correlate MRI changes with clinical progression, monitoring PD symptoms in robust longitudinal clinimetric testing is mandatory [19, 59]. Wearable sensors are increasingly used for this purpose [60]. They show promise in detecting tremor [61], freezing of gait [62], bradykinesia, and dyskinesia [63, 64]. Whether wearables will eventually replace commonly used clinical scoring instruments, such as the MDS-UPDRS remains to be seen [22]. The Personalized Parkinson cohort study in The Netherlands is a parallel longitudinal study in which a wearable sensor will be worn for 24 h a day during a 2 year follow-up period [19]. A subgroup of these participants will also participate in the TRACK-PD study. In the TRACK-PD study, wearables are used at baseline and during follow-up visits in addition to classical MDS scoring scales to measure symptom severity. Data from both studies can potentially be combined in the future, providing an even more complete pallet of information to identify patient subgroups in PD.

A potential limitation of this study is the ability of PD patients to lie still in the scanner for 1 h and the possibility of motion artefacts. However, previous studies have indicated that PD patients are capable and are willing to lie for 1 h in the scanner for obtaining high quality images [65]. Due to the longitudinal nature of this study there is also a risk of high dropout rates during the follow-up period. By including a significant number of participants, we intend to compensate for the expected loss to follow-up. Moreover, by informing them regularly about the study progress, we aim to stay in close contact with all participants and to reduce the amount of loss to follow-up.

## Conclusion

This is the first longitudinal, observational, ultra-high field MRI study in PD patients. With the TRACK-PD study, an important contribution can be made for the improvement of the current diagnostic process in PD. Moreover, this study will be able to provide valuable information related to the different clinical phenotypes of PD and their correlating MRI characteristics. The long-term aim of this study is to better understand PD and develop new biomarkers for disease progression which may help future new therapy development. Eventually, this may lead to predictive models for individual PD patients and towards personalized medicine in the future.

## Data Availability

The datasets generated during this study will become available from the corresponding author on reasonable request.

## Abbreviations

BDI: Beck Depression Inventory
BOLD: Blood oxygen level dependent
CTCM: Clinical Trial Centre Maastricht
EDTA: ethylenediamine tetra-acetic acid
EPI: Echo Planar Imaging
fMRI: Functional MRI
GDPR: General Data Protection Regulation
GRE: Gradient Echo
FoV: Field of view
HC: healthy controls
H&Y: Hoehn and Yahr scale
IMU: Inertial Measurement Unit
LEDD: Levodopa equivalent dose
MDS-UPDRS: Movement Disorders Society Unified Parkinson Disease Rating Scale
MoCA: Montreal Cognitive Assessment
MP2RAGE: Magnetization Prepared 2 Rapid Acquisition Gradient Echoes
MRI: Magnetic Resonance Imaging
MTW TFL: Magnetization transfer weighted TFL
NPA: Neuropsychological assessment
PAS: Parkinson Anxiety Scale
PD: Parkinson’s disease patients
PDQ-8: Parkinson’s Disease Questionnaire
QSM: Quantitative susceptibility mapping
QUIP-RS: Questionnaire for Impulsive-Compulsive Disorders in Parkinson’s disease
RBDSQ: REM-sleep behaviour disorder screening questionnaire
SA2RAGE: Saturation Prepared with 2 Rapid Gradient Echoes
SCOPA-AUT: Scales for Outcomes in Parkinson’s Disease - Autonomic dysfunction questionnaire
TE: Echo time
TI: Inversion time
TR: Repetition time
